# An unwanted intruder

**DOI:** 10.1007/s12471-020-01427-9

**Published:** 2020-05-11

**Authors:** F. F. Gonçalves, L. F. Seca, J. I. Moreira

**Affiliations:** Hospital of Vila Real, Cardiology Department, Centro Hospitalar de Trás-os-Montes e Alto Douro, Vila Real, Portugal

A 79-year-old man with a past history of myocardial infarction and multiple percutaneous coronary interventions was admitted to our coronary care unit with a non-ST-elevation myocardial infarction. Coronary angiography was performed using a 6-Fr hydrophilic sheath through the right radial artery. The diagnostic procedure revealed a new severe focal lesion in the obtuse marginal artery just distal to a previously implanted stent (Fig. 1a). Hence the operator decided to proceed to percutaneous intervention using a left 3.5 extra backup guiding catheter. While the guiding catheter was passing through the radial artery, the patient reported mild discomfort in the forearm and the operator felt some resistance, although it reached the ascending aorta quite easily. After engaging the guiding catheter into the left main coronary artery, with an adequate aortic pressure curve, the patient developed sudden chest pain, and ST elevation was seen on the electrocardiographic monitor. Subsequent contrast injection revealed a total occlusion of the obtuse marginal artery (Fig. 1b).

**Fig. 1:**
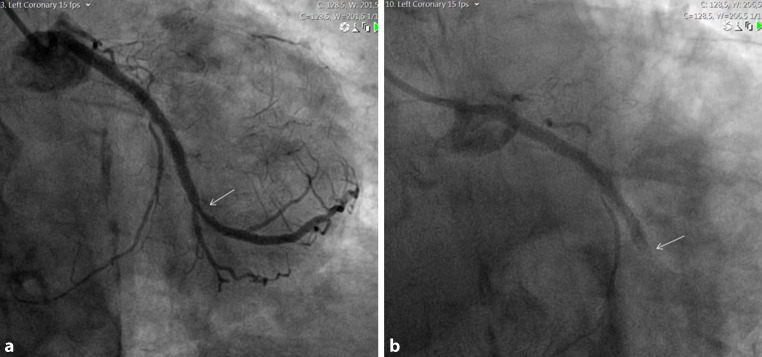
**a** Critical stenosis of the left obtuse marginal artery (*white arrow*). **b** Occlusion of the left obtuse marginal artery (*white arrow*)

What do you think happened and what would you do?

## Answer

You will find the answer elsewhere in this issue.

## Caption Electronic Supplementary Material

Coronary angiograms from the case.

